# Metallic nanoparticles reduce the migration of human fibroblasts in vitro

**DOI:** 10.1186/s11671-017-1982-3

**Published:** 2017-03-17

**Authors:** Larissa Fernanda de Araújo Vieira, Marvin Paulo Lins, Iana Mayane Mendes Nicácio Viana, Jeniffer Estevão dos Santos, Salete Smaniotto, Maria Danielma dos Santos Reis

**Affiliations:** Laboratory of Cell Biology, Institute of Biological Sciences and Health, Federal University of Alagoas, CEP 57072-970 Maceió, Alagoas Brazil

**Keywords:** Silver nanoparticles, Gold nanoparticles, Fibroblast, Extracellular matrix, Cell migration, Cytoskeleton

## Abstract

Nanoparticles have extremely wide applications in the medical and biological fields. They are being used in biosensors, local drug delivery, diagnostics, and medical therapy. However, the potential effects of nanoparticles on target cell and tissue function, apart from cytotoxicity, are not completely understood. Thus, the aim of this study was to investigate the in vitro effects of silver nanoparticles (AgNPs) and gold nanoparticles (AuNPs) on human fibroblasts with respect to their interaction with the extracellular matrix and in cell migration. Immunofluorescence analysis revealed that treatment with AgNPs or AuNPs decreased collagen and laminin production at all the concentrations tested (0.1, 1, and 10 μg/mL). Furthermore, cytofluorometric analysis showed that treatment with AgNPs reduced the percentage of cells expressing the collagen receptor very late antigen 2, α_2_β_1_ integrin (VLA-2) and the laminin receptor very late antigen 6, α_6_β_1_ integrin (VLA-6). In contrast, AuNP treatment increased and decreased the percentages of VLA-2-positive and VLA-6-positive cells, respectively, as compared to the findings for the controls. Analysis of cytoskeletal reorganization showed that treatment with both types of nanoparticles increased the formation of stress fibres and number of cell protrusions and impaired cell polarity. Fibroblasts exposed to different concentrations of AuNPs and AgNPs showed reduced migration through transwell chambers in the functional chemotaxis assay. These results demonstrated that metal nanoparticles may influence fibroblast function by negatively modulating the deposition of extracellular matrix molecules (ECM) and altering the expression of ECM receptors, cytoskeletal reorganization, and cell migration.

**Electronic supplementary material:**

The online version of this article (doi:10.1186/s11671-017-1982-3) contains supplementary material, which is available to authorized users.

## Background

Nanotechnology includes the design, characterization, and application of structures or systems at the nanometre scale (size range, 1–100 nm). Applications related to engineering, information technology, and diagnostics are examples of consumer applications that directly improve our lives. This technology is intrinsically multidisciplinary and is reliant on techniques and methodologies from many fields such as chemistry, physics, electrical engineering, material science, and molecular biology. Our current knowledge of the health effects of nanomaterials is limited and therefore this aspect deserves special attention, particularly the potential long-term adverse effects on living organisms [1, 2].

Metallic nanoparticles exhibit size and shape-dependent properties that are of interest in applications ranging from catalysts and sensing to optics, antimicrobial activity, and data storage [3]. Silver nanoparticles (AgNPs) have been widely used in industrial, household, and healthcare-related products because of their excellent anti-bacterial activity, which is attributed to the strong oxidative activity of AgNP surfaces and the release of silver ions into biological environments [4]. Similarly, the range of uses of gold nanoparticles (AuNPs) in modern medical and biology studies is extremely wide. In particular, it includes genomics, biosensorics, local drug delivery, diagnostics, and medical therapy. With the advances afforded by current research, both types of nanoparticles show promise in several daily applications [5].

In addition to the numerous advantages, it is necessary to know the direct and indirect effects of nanoparticles in cells and tissues. In vitro research is the most appropriate tool for such analysis, for example, on NP toxicity, as studies at the cellular level are simple and convenient than in vivo approaches. However, cytotoxicity may not be the only effect induced by nanoparticles. Morphological changes, cell proliferation, and migration can be affected by nanoparticles [6].

Fibroblasts are a heterogeneous population of structural cells that are distributed on the connective tissue with important role on the deposition of the components of the extracellular matrix (ECM), such as types I, III, and V collagen, and the formation of basement membranes by secreting type IV collagen and laminin [7]. Furthermore, fibroblasts are fundamental in maintaining homeostasis through the secretion of growth factors, release of matrix metalloproteinases, and involvement in wound repair because of cytoskeletal elements that facilitate contraction of healing wounds [8, 9].

We sought to evaluate AuNP and AgNP in vitro cytotoxic effects on human fibroblasts with respect to the ECM production, F-actin cytoskeleton organization, and cell migration.

## Methods

### Cells

The human skin fibroblast cell line CCD­1072Sk was obtained from the Rio de Janeiro Cell Bank (BCRJ; Rio de Janeiro, Brazil). Cells were cultured in RPMI 1640 growth medium supplemented with 10% heat-inactivated foetal bovine serum (FBS), 1% l-glutamine, and 2.5 μg/mL gentamicin (complete medium; all purchased from Invitrogen, CA, USA). Cultures were maintained at 37 °C in a humidified 5% CO_2_ atmosphere. Cells were passaged by trypsin–ethylenediaminetetraacetic acid (EDTA) treatment after every 3–4 days.

### Nanoparticles

Both AgNPs and AuNPs were purchased from Sigma-Aldrich (MO, USA). The AgNPs (cat. no., 730785) were provided in a stabilized suspension in sodium citrate solution at concentration of 0.02 mg/mL, and the AuNPs (cat. no., 752584) were provided in a stabilized suspension in 0.1 mM phosphate-buffered saline (PBS; Sigma-Aldrich). The suspensions were stored at 4 °C until use.

### Cell viability assay

Cells from the CCD­1072Sk line were grown in 96-well plates with complete medium for 16 h until cellular adhesion was attained. The cells were then treated with AgNPs or AuNPs at concentrations of 0.1, 1, or 10 μg/mL for 24 h. After treatment, the cells were incubated with 20 μL of 3-(4,5-dimethylthiazol-2-yl)-2,5-diphenyltetrazolium bromide (5 mg/mL; MTT; Sigma-Aldrich) diluted in 180 μL of RPMI 1640 with 2% FBS. The MTT incubation was carried out for 4 h in a CO_2_ incubator. After this period, the MTT plus medium was completely removed and the reduction of MTT by metabolically active cells was detected by the solubilisation of the formed formazan crystals with 200 μL of dimethyl sulphoxide (DMSO; Sigma-Aldrich). Spectrophotometer readings were obtained at an absorbance of 540 nm (TP-Reader; Thermoplate, Shenzhen, China).

### Immunofluorescence

Fibroblasts (5 × 10^3^) were cultured in 8-well Lab-tek chamber glass slides (Nunc, NY, USA) with complete medium for 16 h to allow cell adhesion. The medium was then replaced and the cells were treated with AgNPs or AuNPs at concentrations of 0.1, 1, or 10 μg/mL for 24 h. Non-treated cells maintained under the same conditions were used as controls. After treatment, the cultures were washed with PBS, fixed with 100% methanol, and subjected to an indirect immunofluorescence assay, as previously described [10]. Briefly, cells were re-hydrated in PBS and incubated for 30 min with PBS containing 1% bovine serum albumin (BSA; Sigma-Aldrich) to block non-specific binding. Next, samples were incubated with primary specific anti-collagen I or anti-laminin (rabbit antibody, 1:50; Sigma-Aldrich) antibodies for 1 h at humidified chamber, washed with PBS, and incubated with goat anti-rabbit-fluorescein isothiocyanate (FITC)–conjugated secondary antibody (1:200, Sigma-Aldrich) for 45 min at room temperature. Immunostained samples were analysed by fluorescence microscopy (Nikon Eclipse 50i; Nikon Instruments Inc., CH, USA). A negative control with secondary antibody was used. Quantitative fluorescence analysis was performed by transformation of specific staining results to pixels and by dividing the total pixel numbers by the area analysed, thus obtaining the number of pixels/μm^2^, using the ImageJ software (NIH, MD, USA).

### Cytofluorometry

Cells previously exposed to AgNPs or AuNPs were incubated with PBS–4% FBS containing a mixture of specific fluorochrome-labelled antibodies used at appropriate dilutions for 20 min at 4 °C, according to the procedure specified by Ayres-Martins et al. [11]. The reagents were as follows: anti-CD49b/PE and anti-CD49f/PE antibodies and the isotype-matched negative controls (all from BD Pharmingen, California, USA). After the cells were stained, they were washed, fixed with 2% formaldehyde, and ultimately analysed by flow cytometry on a FACS Canto II device (Becton Dickinson, California, USA) equipped with the FACS Diva software (Becton Dickinson). A gate excluding cell debris and nonviable cells was determined using forward vs. side scatter parameters. Analyses were performed after recording 10,000–20,000 events for each sample.

### F-actin cytoskeleton staining

The cytoskeleton structure of cultured skin fibroblasts exposed or not exposed to AuNPs or AgNPs was evaluated as previously described [12]. Fibroblasts (2 × 10^3^) were added in a 24-well plate with round glass coverslips previously coated or not coated with fibronectin (10 μg/mL) and incubated for 16 h. After the cells were treated, they were fixed and permeabilized with 4% paraformaldehyde in PHEM solution (60 mM piperazine-1,4-bis(2-ethanesulfonic acid) [PIPES], 2 mM 4-(2-hydroxyethyl)piperazine-1-ethanesulfonic acid [HEPES], 10 mM ethylene glycol tetraacetic acid [EGTA], and 2 mM MgCl_2_; Sigma-Aldrich) containing 0.5% Triton X-100 and 5% sucrose (Sigma-Aldrich). Next, the cultures were stained for F-actin by direct staining with Alexa 488-phalloidin (Molecular Probes, OR, USA) for 1 h. After staining, the coverslips were removed, mounted on permanent slides, and examined using fluorescence microscopy.

### Cell migration assay

Fibroblast migration assays were performed using methods described by Leavesley et al. [13], with some modifications, using transwell inserts with polycarbonate membranes (diameter, 10 mm; pore size, 8.0 μm; Corning Costar, Cambridge, USA). The selection of the pore size was based on previous literature studies which used this type of transwell system to evaluate fibroblast migration [14, 15]. It was also considered the characteristics of the fibroblast cell type, such as their size (10–15 um). Membranes were coated with the blocking reagent 0.1% PBS–BSA for 45 min at 37 °C in 5% CO_2_ humidified atmosphere. After blocking, cells (1 × 10^6^) were added on the upper chamber of the inserts and AgNPs or AuNPs were used as chemotactic factors. Migration was permitted for 24 h at 37 °C in a 5% CO_2_ incubator. After migration, the cells at the bottom of the inserts were stained with 4′,6-diamidino-2-phenylindole (DAPI) and counted using fluorescence microscopy.

### Statistical analysis

The data obtained were analysed using one-way analysis of variance (ANOVA) followed by Newman-Keuls post-test. The values have been represented in terms of mean ± standard error of the mean (SEM) and were considered significant when *p* was ≤0.05.

## Results

### AuNPs and AgNPs impaired ECM deposition in fibroblasts

**Fig. 1:**
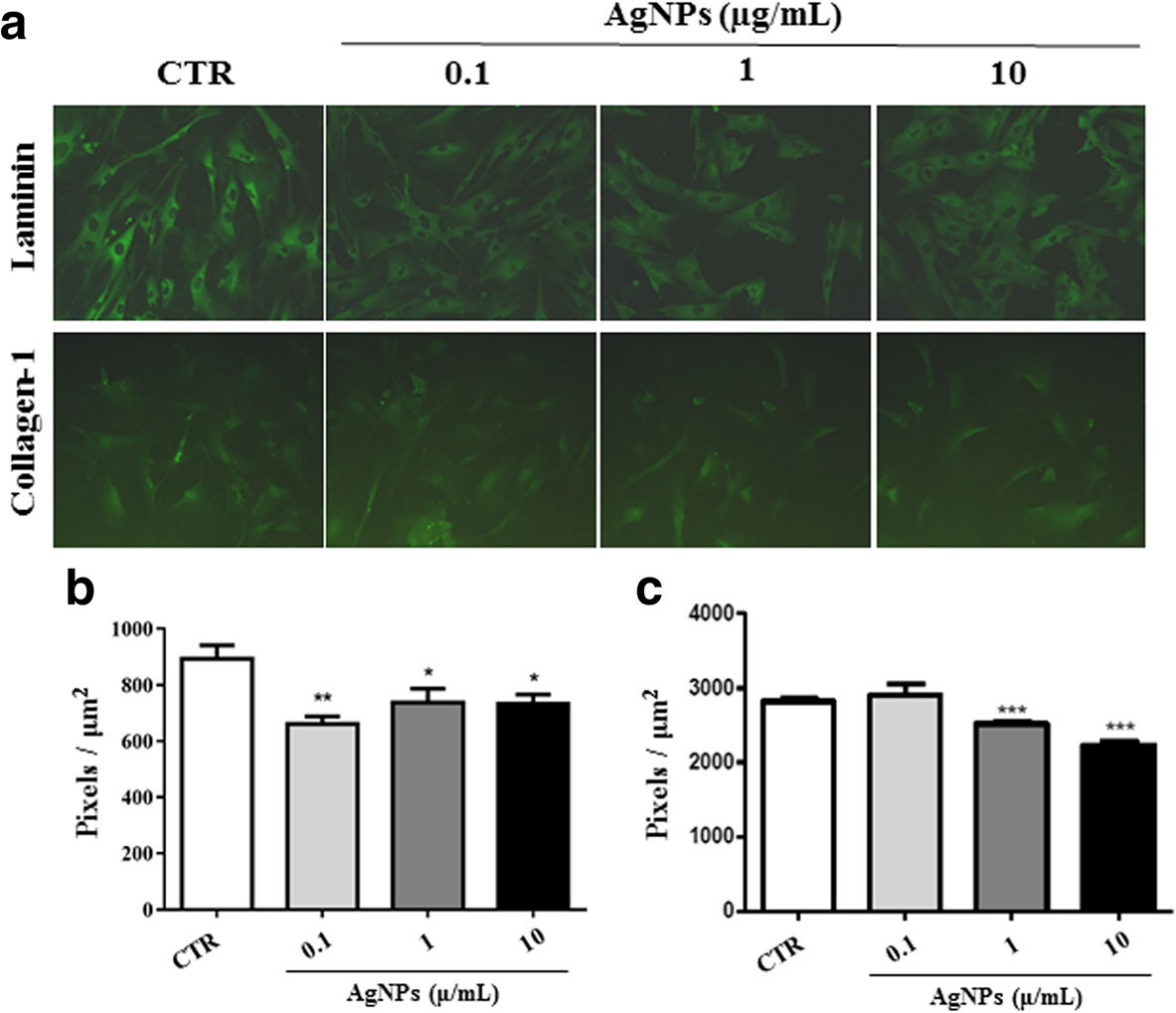
Production of ECM molecules by fibroblasts decreased on exposure to AgNPs. The human fibroblast cell line treated with AgNPs at concentrations of 0.1, 1, or 10 μg/mL for 24 h was analysed using indirect immunofluorescence to detect laminin and collagen-1 production. Photomicrographs in (**a**) show laminin (*green*; *upper panels*) and collagen-1 (*green*; *lower panels*) production in NP-treated cells. Magnification, 200×. Graphs in **b** and **c** show the fluorescence intensity for laminin and collagen-1, respectively. Data have been presented as mean ± SEM. CTR: non-treated cells; *p < 0.05, **p < 0.01, ***p < 0.001

**Fig. 2:**
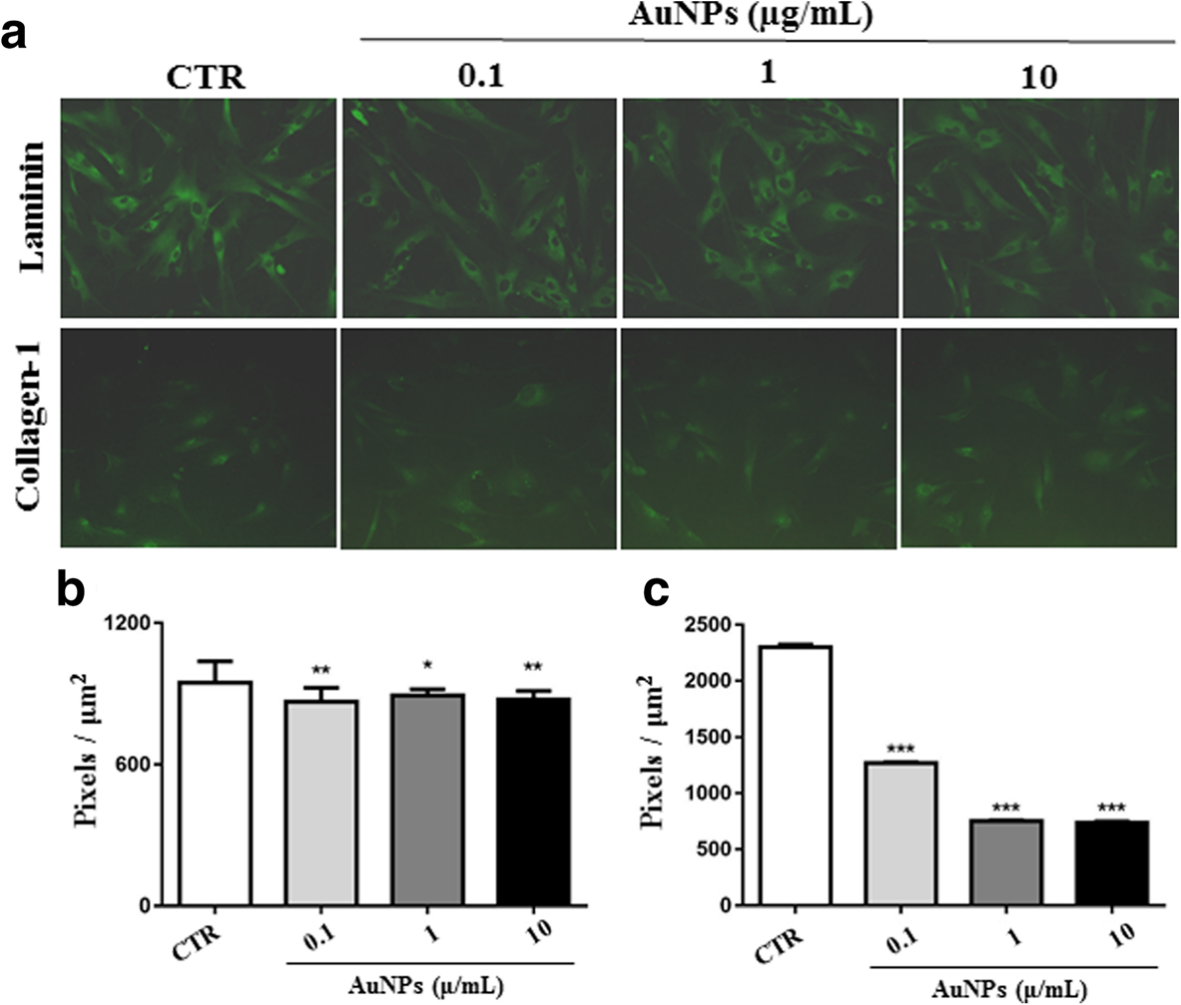
ECM molecule production by human fibroblasts was impaired after exposure to AuNPs. Cells treated with AuNPs at concentrations of 0.1, 1, or 10 μg/mL for 24 h were analysed using indirect immunofluorescence to detect laminin and collagen-1 production. Panels in (**a**) show laminin (*green staining*; *upper panels*) and collagen-1 (*green staining* ; *lower panels*) production in NP-treated cells. Magnification, 200×. *Bar graphs* in **b** and **c** show fluorescence intensity for laminin and collagen-1, respectively. Data have been presented as mean ± SEM. CTR: non-treated cells; *p < 0.05, **p < 0.01, ***p < 0.001

ECM production and deposition in several tissues are normal functions of fibroblasts. Biological conditions (genetic defects or diseases) that involve changes in this functioning of fibroblasts may cause damage to homeostasis of tissues or organs [16]. Our results demonstrate that AgNPs significantly decreased laminin deposition by fibroblasts at all the concentrations tested (0.1: 661.3 ± 27.5; 1: 738.3 ± 50.7; 10: 733.9 ± 33) compared with non-treated cells (CTR: 894.5 ± 48.3) (Fig. 1a, upper panels and 1b). Treatment with AgNPs at 1 (2522 ± 22.9) and 10 μg/mL concentrations (2234 ± 53.9) also decreased collagen deposition in relation to control cells (2820 ± 44,9) (Fig. 1a, lower panels and 1c). The AuNPs, similar to AgNPs, also decreased laminin deposition (CTR: 939 ± 21.7; 0.1: 872.8 ± 15.1; 1: 891.0 ± 6.6; 10: 875.5 ± 8.9) and collagen deposition (CTR: 2300 ± 24.7; 0.1: 1270 ± 8; 1: 753,2 ± 6.8; 10: 736,9 ± 12.3) by fibroblasts after 24 h of treatment (Fig. 2a, upper panels and 2b; Fig. 2a, lower panels and 2c, respectively). When the negative control with secondary antibody was used, it did not show any significant immunolabelling (data not shown).

### ECM receptor expression was altered by NPs

In addition to the ECM molecules produced by fibroblasts, the receptors for these molecules are also expressed on the cell membrane of the fibroblasts. In the activated state, integrins bind to the ECM, signal to the cytoplasm, and evoke cellular responses [17]. After modifications of collagen and laminin deposition, we investigated the effects of NPs on the expression of the main integrin-type receptors for these molecules, very late antigen 2, α_2_β_1_ integrin (VLA-2) and very late antigen 6, α_6_β_1_ integrin (VLA-6), by analysing their alpha chains (CD49b and CD49f, respectively) through flow cytometry. AgNPs caused reduction in the percentage of cells expressing both integrin alpha chains on the fibroblast membrane (Table 1). Similarly, AuNPs decreased the percentage of cells with CD49f expression (Table 1). In contrast, treatment with 1 μg/mL of AuNPs increased the percentage of cells expressing the CD49b chain (Table 1).

**Table 1:** Percentage of cells expressing VLA-2 and VLA-6 on their surface on NP exposure

Groups	VLA-2 (CD49b)	VLA-6 (CD49f)
*AgNPs*		
CTR	66.03 ± 1.50	22.78 ± 0.84
0.1 μg/mL	54.28 ± 1.14**	20.31 ± 0.42*
1 μg/mL	58.57 ± 1.54*	19.99 ± 0.45*
10 μg/mL	47.69 ± 0.51***	19.82 ± 0.33*
*AuNPs*		
CTR	64.35 ± 1.28	19.56 ± 0.43
0.1 μg/mL	79.17 ± 5.67	17.92 ± 0.37*
1 μg/mL	80.39 ± 0.98***	17.55 ± 0.25*
10 μg/mL	61.67 ± 1.38	17.31 ± 0.26**

CTR: non-treated cells; *p < 0.05; **p < 0.01; *** *p* < 0.001

### Cytoskeletal reorganization was induced by NPs

**Fig. 3:**
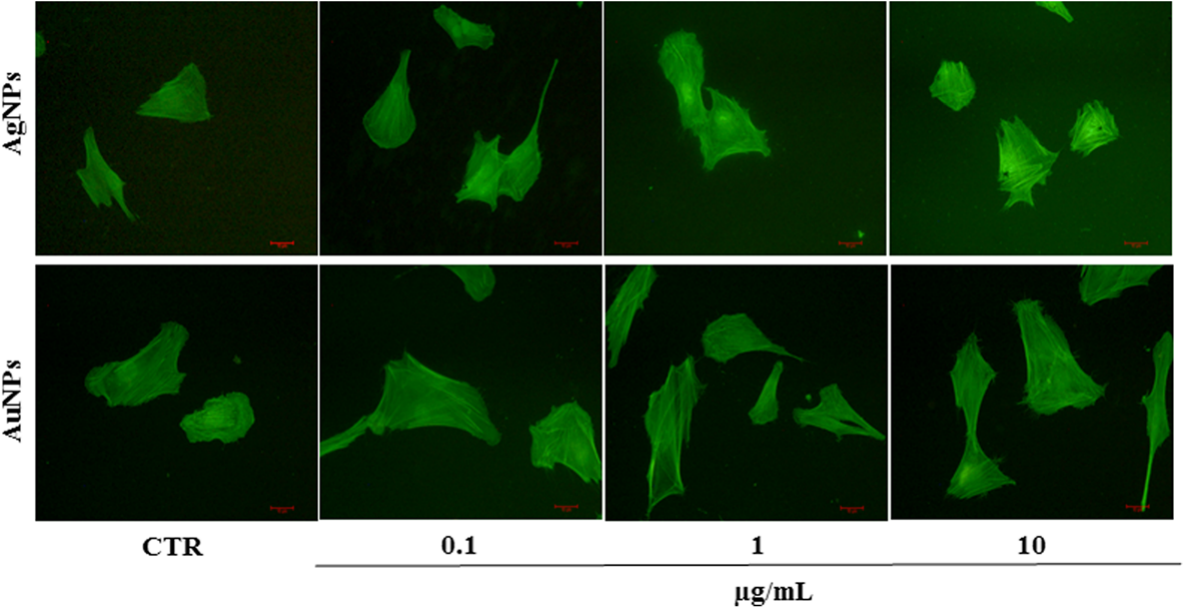
Cytoskeletal reorganization in human fibroblasts treated with NPs. Cells exposed to AuNPs and AgNPs at 0.1, 1, or 10 μg/mL for 24 h were stained with phalloidin to analyse F-actin distribution (*green staining*). Photomicrographs revealed alterations in cell polarity and stress fibres in NP-treated cells. CTR: non-treated cells. Magnification, 200×

The results described so far indicate that NPs may be involved in ECM function. Therefore, we aimed to study possible cytoskeleton changes under NP exposure because cytoskeleton molecules also interact with the ECM to permit cell adhesion and migration. For this, previously NP-exposed fibroblasts were stained with F-actin-specific Alexa 488–conjugated phalloidin. Increase of actin fibres was observed in all AgNP-treated fibroblasts when compared with control cells, accompanied with loss of cell polarization. Fibroblasts treated with the three studied concentrations of AuNPs exhibited an increase in cell spreading, as compared to that noted for non-treated cells (Fig. 3).

### NPs reduced in vitro fibroblast migration

**Fig. 4:**
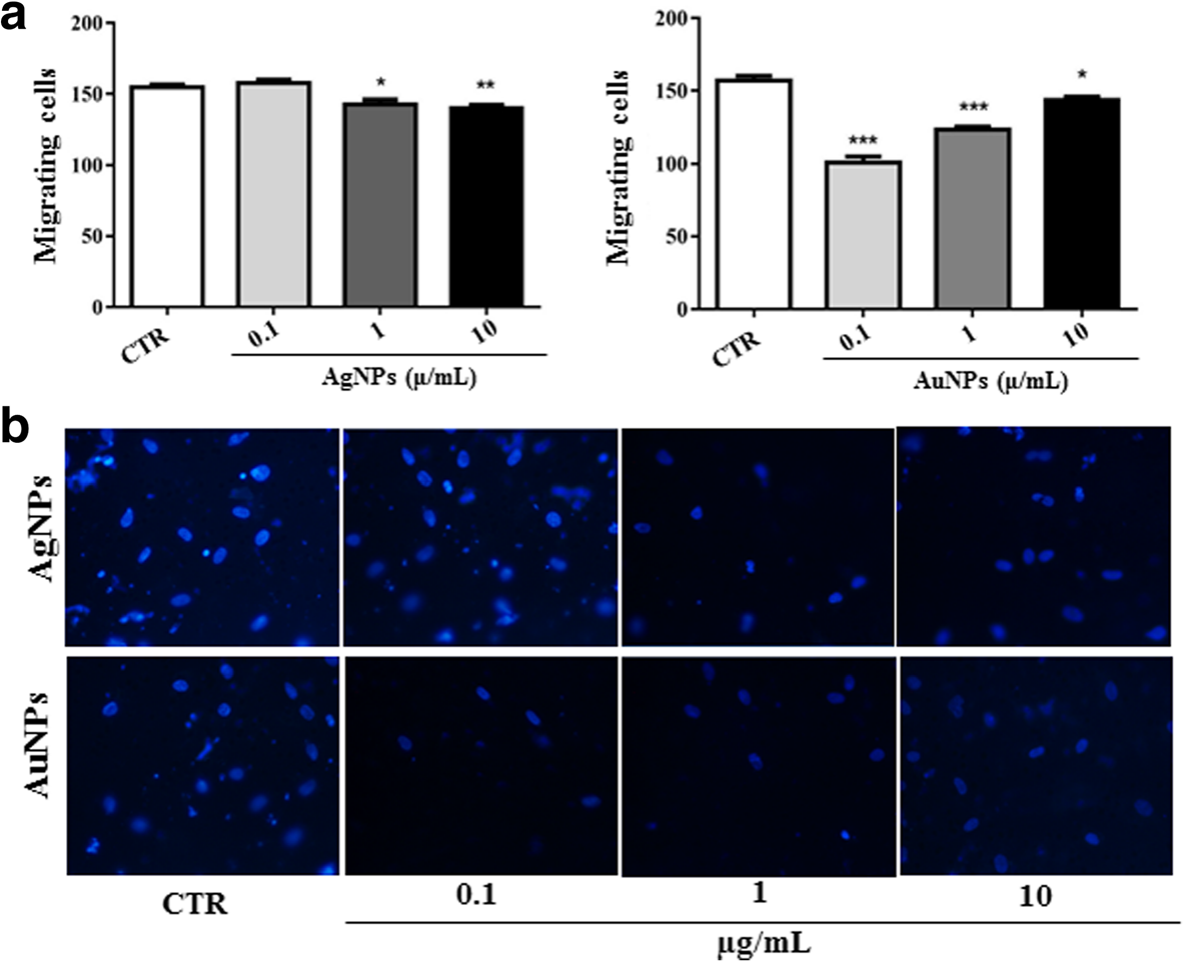
NPs reduced human fibroblast migration in vitro. AgNPs and AuNPs were used as chemotactic factors for fibroblasts in transwell migration assays. Cells were permitted to migrate for 24 h at 37 °C in a 5% CO_2_ incubator. Fibroblasts allowed to migrate through medium alone were used as controls (CTR). After migration, the cells at the bottom of the inserts were stained with DAPI and counted using fluorescence microscopy. *Bar graphs* in **a** show the mean ± SEM values for the number of migrating cells. *p < 0.05, **p < 0.01, ***p < 0.001. Representative photomicrographs of the DAPI-stained nucleus (*blue staining*) used for quantification of migrating cells are shown in **b**. Magnification, 200×

Since the NPs studied herein altered both the fibroblast ECM and cytoskeleton, we hypothesized that exposure to this material would affect fibroblast motility. Therefore, we evaluated the migration of these cells on transwell inserts. Both AuNP and AgNP treatments were found to reduce the number of migrating cells. The reduction was observed on fibroblasts treated with 1 (143.0 ± 3.1) and 10 μg/mL (140.3 ± 2.5) of AgNPs compared with non-treated fibroblasts (154.5 ± 2.3) (Fig 4a, left graph and 4b, upper panels). All concentrations of AuNPs reduced fibroblast migration (CTR: 157.0 ± 3.3; 0.1: 100.8 ± 4.3; 1: 123.5 ± 2; 10: 143.8 ± 2.2), but the decrease was more pronounced for the 0.1 μg/mL concentration of AuNPs (Fig. 4a, right graph and 4b, lower panels).

### NPs did not alter cell viability

**Fig. 5:**
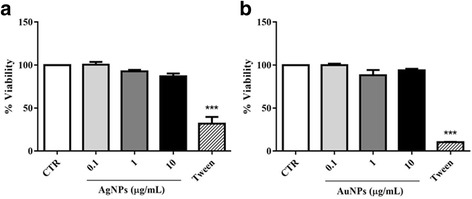
NP exposure did not alter fibroblast viability. After the human fibroblasts were treated with AgNPs and AuNPs, they were analysed using the MTT assay to measure cell viability as described in the Methods section. Cells treated with Tween were used as the positive control for cell death and cells cultured with medium alone were used as controls (CTR). The graphs show the cell viability percentage for AgNP-treated cells (**a**) and AuNP-treated cells (**b**) and their respective controls. Data are presented as mean ± SEM

The MTT assay was performed to verify if the impairment of function induced by NPs in fibroblasts could be due to interference with cell viability. Both AuNP and AgNP treatments did not alter fibroblast viability (Fig.5; Additional file 1). This result indicates that AuNPs and AgNPs may influence important aspects of in vitro fibroblast function and that this impairment does not involve cellular alterations related to metabolism and cell viability.

## Discussion

The development of nanoparticles for biomedical applications, including medical imaging and drug delivery, is currently undergoing dramatic expansion. However, as the range of nanoparticle types and applications increases, it is also clear that the potential toxicities of these novel materials and the properties driving such toxic responses must also be understood [18]. Thus, unfavourable tissue reactions promoted by NPs, such as degeneration, necrosis, inflammation, tissue fibrosis, or even the induction of tumour growth, have been the target of monitoring [19–22]. In the current study, AgNPs and AuNPs promoted toxicological effects on fibroblasts, as shown by decreased ECM deposition, imbalanced expression of integrins, impaired F-actin cytoskeletal reorganization, and decreased human fibroblast migration, without affecting cell viability.

Human dermal fibroblast cells are found throughout the human body and are responsible for the maintenance of soft tissue integrity through their primary function, which is collagen expression. Collagen is the most abundant protein in the ECM [23] and provides binding sites for adhesion molecules involved in cell attachment [24–26], such as those for direct association between collagen IV and laminin [27]. In the current study, it was found that collagen and laminin deposition on fibroblasts was decreased by treatments with all concentrations of AgNPs and AuNPs, except for 0.1 μg/mL AgNP, which did not alter collagen deposition. This result is in agreement with similar study in that human fibroblast cells showed 20 and 95% reduction of collagen production following exposure to AuNPs and AgNPs, respectively [28]. Other studies have shown that protein synthesis for ECM formation is altered dramatically and that significantly lesser collagen and fibronectin are expressed by cells exposed to AuNPs [6, 29]. In previous studies, treatment with titanium dioxide NP (TiO_2_) also decreased the ability of human fibroblasts to cause collagen contraction [30] and to induce procollagen-1 repression [31].

To better understand the basis of decrease in ECM proteins, integrins, which are cell surface proteins that bind to ECM molecules to mediate cell–cell adhesion [32], were evaluated. Collagen and laminin integrin receptor expression was determined by evaluating VLA-2 integrin alpha chain (α2; CD49b) and VLA-6 integrin alpha chain (α6; CD49f) because the main receptors for the different collagen types are α1β1 (VLA-1) and α2β1 (VLA-2) integrins [33, 34] and the receptor involved in the adhesion of several cell types to laminin is the α6β1 (VLA-6) integrin [35, 36]. In our current study, we found that incubation with AgNPs markedly decreased the percentage of cells expressing the α2 chain and the α6 chain, representing the VLA-2 and VLA-6 integrins, respectively. A similar decrease was also observed on VLA-6-expressing cells after AuNP exposure. Use of 1 μg/mL AuNPs increased the percentage of VLA-2-expressing fibroblasts, whereas AuNP treatment at 0.1 and 10 μg/mL concentrations did not affect the expression of this receptor. Currently, the reasons for the increase in VLA-2-expressing cells after AuNP incubation are unclear. A possible explanation is that the increase or decrease in integrin expression may not directly affect cell function but may affect the activation state of the receptors, as seen in the study by Smaniotto et al. [10]. In that study, thymocytes from growth hormone-transgenic mice showed increased adhesion and migration through laminin substrate, but VLA-6 expression did not change significantly, indicating an increase in the activation levels of this integrin without increase in the expression of the corresponding protein. Therefore, it is necessary to perform more studies on intracellular signalling to understand the mechanisms underlying the effects of AuNPs on VLA-2 expression on fibroblasts.

Disruption of the expression of ECM proteins may responsible for the lack of tension in the actin fibres because these fibres would be anchored to integrins. Integrins bound to Arg-Gly-Asp (RGD) domains found on secreted ECM proteins, which are primarily collagen and fibronectin in the case of fibroblasts [29]. When the F-actin cytoskeleton was analysed, imbalanced effects were found for fibroblasts treated with AgNPs and AuNPs. Cells treated with AgNPs showed a non-polarized shape, in contrast to that noted in tissue culture, in that many cells can adopt a polarized morphology that allows them to move through the polarized and dynamic reorganized actin cytoskeleton [37]. Additionally, stress fibre formation, pivotal to maintaining the polarized cell structure [38, 39], also increased after AgNP treatments. This impairment was similar to that observed in fibroblast mutants that lacked the glycoprotein transmembrane SHPS-1 and exhibited increased formation of actin stress fibres and focal adhesions but were defective in subsequent polarized extension [40]. Previous studies also demonstrated the impact of NP exposure on fibroblast cytoskeleton, such as the filament disruption and actin stress fibre disappearance promoted by AuNPs [6, 31], and induced repression of α-actin expression by TiO_2_ NPs [29]. In our study, AuNPs exposure led to increase in cell spreading, without significant changes in cell shape and polarization.

In directed cell migration, spatial asymmetry necessary for early events that establish polarized leading and trailing edges is acquired [41]. Cell migration was performed under chemotactic effects of AgNPs and AuNPs, but this process was completely impaired by nanoparticle treatments because the fibroblast migration capacity was dramatically reduced by AgNPs at concentrations of 1 and 10 μg/mL and by AuNPs at all concentrations. Similarly, TiO_2_NP also decreased fibroblast mobility and impaired cell function, with the latter effect being more potent in leading to damage [30]. A possible mechanism for reduction of migration is the increased formation of intracellular stress fibres, which is correlated with enhanced cell adhesion; mammary migrating cells have fewer stress fibres [39, 42]. Furthermore, studies have shown that decrease in cellular motility is also induced by AuNPs because of abnormal formation of actin filaments on dermal fibroblasts [6, 43]. Although fibroblast migration has been shown to involve α2β1 integrin [44, 45], the increase in VLA-2 expression due to exposure to 1 μg/mL AuNPs could not prevent reduction of migration. This is consistent with emerging evidence showing that increase or decrease in integrin expression may not directly affect migration, as shown by Nagib et al. [46]. In that study, thymocytes from diabetic mice that expressed lower VLA-5 and VLA-6 levels did not show altered migration ability in vitro, indicating that only lower integrin expression on the cell surface is not sufficient to hinder migration.

Despite the cytotoxic effects of AgNPs and AuNPs with respect to fibroblast function, these cells were not sensitive to the tested concentrations of both AgNPs and AuNPs in the MTT assay. These results are consistent with previous study performed with PC-3 and MCF-7 cells, which reported that AuNPs coated with citrate, starch, and Arabic gum, and studied using different cytotoxicity assays (e.g., MTT assay, neutral red, and lactate dehydrogenase release) showed excellent cell viability, indicating that AuNPs were nontoxic to the array of cells tested [43] (Additional File 1). Also, it was shown that AuNPs can alter cell function without modifications on cell viability, corroborating with our findings. It was observed that macrophages from RAW 264 cell lines exposed to 100–400 μg/ml of AuNPs stabilized with polyvinylpyrrolidone (PVP), with approximately 36 nm of diameter and spherical shape inhibited metalloproteinase (MMP) activity, while they did not alter cell cytotoxicity [47]. The same group showed that these AuNPs did not alter fibroblast viability, while they could inhibit MMP-1 activity [48]. It is possible that the effect on cell viability may be related with NP size, since human dermal fibroblasts treated with 30, 50, and 90 nm AuNPs have a decrease on cell viability in a dose-dependent manner, with a reduction in cell survival at concentrations up to 5 μg/ml [49]. In this case, the reduction was observed after 48 h. In contrast, the AgNPs induced marked adverse effects on cell viability and could induce changes in cell shape, decrease cell viability, increase lactate dehydrogenase release, and finally cause cell apoptosis and necrosis [3, 6, 50, 51].

## Conclusions

Our findings demonstrate that both AgNPs and AuNPs can impair fibroblast function and cause decreased ECM deposition, imbalanced integrin expression, impaired F-actin cytoskeletal reorganization, and decreased cell migration, without loss of cell viability. These results highlight the importance of performing biocompatibility studies using different approaches besides from cell viability to better understand the effects of NPs on their target structures before they can be safely applied in clinical trials.

## Additional file

Additional file 1: Figure S1. Effects of NP exposure on fibroblast viability. After the human fibroblasts were treated with AgNPs and AuNPs, they were analysed using the MTT assay to measure cell viability. Cells treated with Tween were used as the positive control for cell death and cells cultured with medium alone were used as negative controls (CTR). The graphs show the cell viability percentage for AgNP-treated cells (a) and AuNP-treated cells (b) and their respective controls. Data are presented as mean ± SEM. (DOCX 47 kb)

## Abbreviations

FBS: Foetal bovine serum
FITC: Fluorescein isothiocyanate
MTT: 3-(4,5-dimethylthiazol-2-yl)-2,5-diphenyltetrazolium bromide
PBS: Phosphate-buffered saline
PE: Phycoerythrin
RPMI: Roswell Park Memorial Institute
TiO_2_: Titanium dioxide
VLA-2: Very late antigen 2, α_2_β_1_ integrin
VLA-6: Very late antigen 6, α_6_β_1_ integrin
