# Supplementary material for: Metallic nanoparticles reduce the migration of human fibroblasts in vitro
Source: Nanoscale Res Lett. 2017 Mar 17;12:200. doi: 10.1186/s11671-017-1982-3 (PMC5355407; doi:10.1186/s11671-017-1982-3)
Supplement: Additional file 1: Figure S1. — Effects of NP exposure on fibroblast viability. After the human fibroblasts were treated with AgNPs and AuNPs, they were analysed using the MTT assay to measure cell viability. Cells treated with Tween were used as the positive control for cell death and cells cultured with medium alone were used as negative controls (CTR). The graphs show the cell viability percentage for AgNP-treated cells (a) and AuNP-treated cells (b) and their respective controls. Data are presented as mean ± SEM. (DOCX 47 kb) [file 11671_2017_1982_MOESM1_ESM.docx]

**Additional file**

**
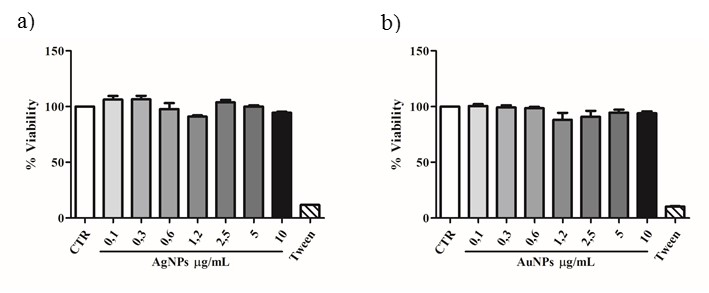
**

b)

a)

**Figure 1. Effects of NP exposure on fibroblast viability.** After the human fibroblasts were treated with AgNPs and AuNPs, they were analysed using the MTT assay to measure cell viability. Cells treated with Tween were used as the positive control for cell death and cells cultured with medium alone were used as negative controls (CTR). The graphs show the cell viability percentage for AgNP-treated cells (a) and AuNP-treated cells (b) and their respective controls. Data are presented as mean ± SEM.
